# Eating Disorders During Emerging Adulthood: A Systematic Scoping Review

**DOI:** 10.3389/fpsyg.2019.03062

**Published:** 2020-01-31

**Authors:** Rachel Potterton, Katie Richards, Karina Allen, Ulrike Schmidt

**Affiliations:** ^1^Section of Eating Disorders, Institute of Psychiatry, Psychology and Neuroscience, King's College London, London, United Kingdom; ^2^The Eating Disorders Service, Maudsley Hospital, South London and Maudsley NHS Foundation Trust, London, United Kingdom; ^3^School of Psychological Science, The University of Western Australia, Crawley, WA, Australia

**Keywords:** eating disorders, anorexia nervosa, bulimia nervosa, binge eating disorder, emerging adulthood

## Abstract

**Background:** Eating disorders (EDs) during the transition to adulthood can derail social, psychological, and vocational development. Effective treatment is of paramount importance, yet young adults' treatment needs are typically less well met than those of adolescents. In recent years, there has been a considerable shift in how developmental psychologists understand the transition to adulthood, with this life-phase reconceptualized as “emerging adulthood” (EA) (~18–25 years). Engagement with burgeoning developmental research is likely key to providing more effective care for young people experiencing EDs.

**Aims:** To review ED research which has utilized the concept of EA, and to assess the usefulness of this concept for ED research and practice.

**Methods:** A systematic scoping review was conducted in accordance with the Joanna Briggs Institute guidelines for scoping reviews. Three databases (Psychinfo, PubMed, Embase) were searched for papers which explicitly focused on EDs during EA. No restrictions as to publication type, language, study design, or participants were applied. Included studies were assessed for developmental “informedness,” and findings were qualitatively synthesized.

**Results:** Thirty-six studies (*N* = 25,475) were included in the review. Most studies used quantitative methodologies, were cross-sectional in design and focused on identifying psychological and social factors which contribute to etiology of EDs. Many studies (*N* = 22) used well-defined samples of emerging adults (EAs); few studies (*N* = 8) included developmental measures relevant to EAs. Findings indicate that whilst factors implicated in EDs in adolescence and adulthood are relevant to EAs, EA-specific factors (e.g., identity exploration) may also contribute. Conventional ED services and treatments present difficulties for EAs, whilst those adapted to EAs' needs are feasible, acceptable, and more effective than treatment-as-usual. Directions for future research and clinical implications are discussed.

**Conclusion:** Existing research indicates that the EA concept is relevant for understanding EDs during the transition to adulthood, and ED services should implement adaptations which exploit the opportunities and overcome the challenges of this developmental stage. EA is currently an underused concept in ED research, and future engagement with the developmental literature by both researchers and clinicians may be key to understanding and treating EDs during transition to adulthood.

## Introduction

“*I would there were no age between ten and three-and-twenty, or that youth would sleep out the rest; for there is nothing in the between but getting wenches with child, wronging the ancientry, stealing, fighting.” (A Winter's Tale, William Shakespeare)*.

### Eating Disorders During Transition to Adulthood

Eating disorders (EDs), including anorexia nervosa (AN), bulimia nervosa (BN), and binge eating disorder (BED), are serious mental illnesses characterized by disturbances of body image and eating behavior (American Psychiatric Association, 2013). EDs typically have their onset during the transition to adulthood; mean age of onset for AN and BN is between 15 and 19 years, whilst BED typically occurs slightly later, between 23 and 24 years (Hudson et al., 2007; Kessler et al., 2013; Micali et al., 2013; Steinhausen and Jensen, 2015). Young adults' treatment needs are less well met than those of adolescents, as indicated by lower rates of access, increasing hospital admissions, treatment dissatisfaction, disengagement and poorer clinical outcomes (Weigel et al., 2014; Care Quality Commission, 2018; Mitrofan et al., 2019).

Restrictions in access to specialist care undoubtedly contribute to heightened vulnerability and unmet need during the transition to adulthood. In the United Kingdom (UK), standards specifying maximum waiting times of four weeks for ED treatment only apply to children and adolescents, and individuals aged 18 years or over wait longer than those aged under 18 years for treatment (NHS England, 2015; Beat, 2018; Royal College of Psychiatrists, 2019). Furthermore, long waiting times appear to have a more negative impact on 18 to 25-year-olds than older patient groups (Sánchez-Ortiz et al., 2010). Many young people begin university during this time, and mismanagement of resultant care transitions likely contributes to patient disengagement, deterioration and—in extreme cases—death, as highlighted by a recent UK government report (Parliamentary and Heath Service Ombudsman, 2017). However, other factors are also likely to contribute. In particular, there has been significant recent progress in basic developmental research with regards to understanding the transition to adulthood, and engagement with this research may be key to better understanding and treating EDs during this life-phase (Blakemore, 2018).

### Developmental Conceptualizations of Transition to Adulthood

Recent years have seen a considerable shift in how developmental psychologists understand the transition from childhood to adulthood (Ledford, 2018). Historically, both developmental psychologists and lay people have understood that adulthood is achieved at or close to an individual's 18th birthday (Dahl et al., 2018). Indeed, the 16th and 18th birthdays are associated with the attainment of increased legal rights and responsibilities (e.g., age of sexual consent, acquiring a driving license, purchasing cigarettes and alcohol, voting in elections, joining the army) (Dahl et al., 2018). However, recent improvements in brain imaging technologies have made it increasingly apparent that the human brain is not fully developed until the twenties (Dahl et al., 2018). Additionally, social and economic changes (e.g., increased access to third-level education; housing costs; acceptance of extra-marital sex/cohabitation; improvements in reproductive health) have meant that many of the key milestones of adulthood (e.g., marriage, parenthood, home ownership) are being attained much later than in previous decades (Ledford, 2018; Office of National Office for National Statistics, 2018). Accordingly, there has been a growing consensus amongst developmental researchers that adulthood is not achieved until the mid-twenties (Steinberg, 2014; Blakemore, 2018; Ledford, 2018).

### A New Conceptualization: Emerging Adulthood

Less agreement exists as to how to best to characterize the newly prolonged transition to adulthood. Some researchers conceptualize this development as an “extended adolescence” (Steinberg, 2014; Blakemore, 2018). Others have suggested that, in Western cultures, this should be considered as a stand-alone developmental stage called “emerging adulthood” (EA) (Arnett, 2000). EA is defined as the period between when a person leaves secondary school and when they attain adult roles (~18–25 years of age) (Arnett, 2000). Whilst there is some overlap with adolescence in terms of developmental tasks, EA is understood to be associated with a pattern of psychological characteristics, brain development and social context distinct from both adolescence (~12–18 years) and young adulthood (~25–40 years) (Arnett, 2000).

EA is associated with an intensification of both autonomy and identity development relative to adolescence (Phinney et al., 2005; Luyckx et al., 2006, 2008; Klimstra et al., 2010; Schwartz et al., 2013; Inguglia et al., 2015; Verschueren et al., 2017b). This intensification is likely due in part to EAs' unique social context which facilitates such developmental processes. EAs tend to exist outside prescribed social roles; they have few of the restrictions of adolescence (e.g., parental supervision, legally restricted access to substances) and few of the responsibilities of adulthood (e.g., work, children, financial obligations) (Arnett, 2000). Indeed, EAs report that they feel in-between childhood and adulthood, that they have few obligations toward others, and that there are many life-paths open to them (Nelson and Barry, 2005; Sirsch et al., 2009; Arnett and Padilla-Walker, 2015; Wängqvist and Frisén, 2015; Arnett and Mitra, 2018). Accordingly, EAs experience more demographic change (e.g., frequent residence, occupation and relationship changes) and are a far more demographically heterogenous population than both adolescents and young adults (Arnett, 2000; Department for Communities and Local Government, 2016; Frances-Devine, 2019).

EA is also associated with patterns of structural and functional brain development distinct from those seen during adolescence (Taber-Thomas and Pérez-Edgar, 2015). Neuroimaging studies have found that cerebral cortex development occurs in a “back-to-front” direction, such that the prefrontal cortex (PFC) is the focus of development during the EA years (Giedd et al., 1999; Sowell et al., 1999; Gogtay et al., 2004; Paus, 2005; Shaw et al., 2008; Raznahan et al., 2011). The PFC is associated with a range of executive functions, including working memory, planning and self-monitoring, and performance on measures of these abilities (e.g., Stroop Task; Tower of London task) continues to improve steadily throughout adolescence and EA, plateauing between the ages of 23 and 26 (Steinberg et al., 2018). Projection fibers between the PFC and subcortical structures (e.g., the striatum) also continue to develop into the twenties (Liston et al., 2005; Ashtari et al., 2007; Bonekamp et al., 2007; Tamnes et al., 2009; Asato et al., 2010). Connections between the PFC and subcortical areas are believed to be instrumental in decision-making and goal-directed behavior (Yuan and Raz, 2014). Indeed, performance on measures of decision-making (e.g., Stoplight task, Iowa gambling task) gradually improves across the course of EA (Steinberg et al., 2018), and is associated with differential patterns of brain activation in EAs compared to adolescents (Bjork et al., 2004; Ernst et al., 2005; Galvan et al., 2006; Van Leijenhorst et al., 2009).

### Eating Disorders During Emerging Adulthood

Many people experience EA as a positive and exciting time (Arnett, 2000). However, mental illness is also prevalent during this life-stage (Kessler et al., 2005). It may be that ongoing physical, psychological and social development contributes to the onset and maintenance of a wide range of mental illnesses, including EDs, during EA (McGorry et al., 2014; Taber-Thomas and Pérez-Edgar, 2015; Blakemore, 2018, 2019). Additionally, EAs are usually treated in adult mental health services and incompatibility between the distinctive developmental needs of EAs and the culture of adult services may contribute to reluctance to access, dissatisfaction, disengagement, and poor clinical outcomes (McGorry et al., 2014). This incompatibility is particularly relevant to ED services, as there is a clear shift in treatment philosophy—relating to how personal responsibility is understood and managed—in services for under 18s compared to ED services for 18 years and over (Winston et al., 2012). Despite this apparent relevance, it is not clear to what extent the concept of EA has been integrated into the ED field.

### Aims of the Review

This paper aimed to review existing ED research which has explicitly utilized the concept of EA, with a view to answering the following questions:

- What are the characteristics of these studies (e.g., country of origin; sample; design)?
- What are the aims of these studies (e.g., investigating prevalence; etiology; treatment)?
- To what extent could these studies be considered to be informed by EA-focused developmental research?
- What have these studies found?

## Methods

A systematic scoping review methodology was used to review existing research into EDs during EA. This methodology was deemed appropriate as this is a new and heterogeneous research area, and scoping reviews aim to determine the extent and nature of available research in fledgling and diverse fields (Peters et al., 2015). Scoping reviews therefore usually include a broader range of evidence sources (e.g., conference abstracts; unpublished dissertations) than conventional systematic reviews. This review was conducted in accordance with the guidelines for scoping reviews developed by the Joanna Briggs Institute (Peters et al., 2017) and the PRISMA statement guidelines for scoping reviews (Tricco et al., 2018).

### Search Strategy

Three databases (Psychinfo, PubMed, Embase) were searched for papers published from database inception until 22nd May 2019. The following search terms were used: (eating disorder^*^ OR anorexi^*^ OR bulimi^*^ OR binge eat^*^ OR disordered eat^*^; *title/abstract*) AND (emerging adult^*^; *title/abstract*). Database searches were supplemented by internet searches, and the reference lists of included studies were also hand-searched for additional relevant papers.

### Selection Process

Prior to study selection, eligibility criteria for study objective and methodology were specified (see Table 1). No restrictions were applied for publication type, language, design, or sample characteristics. Titles and abstracts of the retrieved papers were pre-screened independently by two reviewers (RP and KR) using the eligibility criteria. Full texts were then screened independently by the same reviewers. All papers that did not meet the eligibility criteria were excluded, and reasons for their exclusion documented. Discrepancies between the reviewers regarding inclusion and exclusion decisions were resolved through discussion.

**Table 1:** Systematic scoping review eligibility criteria.

	**Included**	**Excluded**
Publication type	Peer-reviewed journal articles Book chapters Conference abstracts Unpublished dissertations	None
Language	Any	None
Study objective	Explicit focus on eating disorders during emerging adulthood	No explicit focus on eating disorders during emerging adulthood
Methodology	Quantitative Qualitative Mixed methods	Narrative reviews Systematic reviews Meta-analyses
Design	Any	None
Sample	Any	None

### Data Charting and Analysis

Relevant data (country of origin; publication type; study objective; methodology; design; sample characteristics; relevant findings) were extracted from the included papers using a pre-piloted data form by one reviewer (RP). These data were checked by a second reviewer (KR).

#### Categorization of Study Focus

From data pertaining to study objective, six categorizations of study focus were devised (see Table 2), and each paper categorized by one reviewer (RP). For each study focus category, extracted data pertaining to publication type, country of origin, methodology, design and sample were summarized using descriptive statistics.

**Table 2:** Study focus categorization system.

**Categorization of study focus**	**Example research question**
Prevalence	To analyse the prevalence of eating disorders in emerging adults
Impact	To examine the impact of eating disorders during emerging adulthood on outcomes during young adulthood
Trajectory	To characterize the longitudinal stability of eating disorders from adolescence to emerging adulthood
Etiology	To examine the role of psychological, social, and biological factors in eating disorders during emerging adulthood
Treatment	To assess clinical outcomes in patients who have received treatment adapted to emerging adults
Multiple	Any combination of two or more of the above research questions

#### Rating of Developmental “Informedness”

The extent to which each study was informed by existing EA-focused developmental research was assessed independently by two reviewers (RP and KR), using a rating system devised by the authors. Studies were rated as “strong” if they included developmental indices understood to be relevant to EAs (Wagner, 2008) (see Table 3 for list of developmental indices, based on existing developmental literature). Studies were rated as “moderate” if they did not include developmental indices but did include a clearly defined sample of EAs only (mean age and/or age-range between 18 and 25 years), as well as a clear rationale of why this EA sample was chosen. Whilst age is often used as a proxy for developmental level, it is not synonymous with developmental stage and is therefore less optimum than direct measures of development (Wagner, 2008). Studies were rated as “weak” if they did not include developmental indices and did not include a clearly defined sample of EAs only. Discrepancies between the reviewers regarding rating decisions were resolved through discussion.

**Table 3:** Developmental processes during emerging adulthood of putative relevance to eating disorders.

**Process**	**Example indices**
Maturation of prefrontal cortex and connections with limbic system	Magnetic resonance imaging Functional magnetic resonance imaging Diffusion tensor imaging
Identity development	Self-report questionnaires (e.g., DIDS, UMICS)
Autonomy development	Parent-report and self-report questionnaires (e.g., AFC; EAS)
Decision-making	Experimental paradigms (e.g., Stoplight task) Self-report questionnaires (e.g., SSS)
Role transitions (e.g., educational, residential)	Self-report questionnaires (e.g., LEDS)

*AFC, Autonomous Functioning Checklist; DIDS, Dimensions of Identity Development Scale; EAS, Emotional Autonomy Scale; LEDS, Life Events and Difficulties Schedule; SSS, Sensation Seeking Scale; UMICS, Utrecht-Management of Identity Commitments Scale*.

#### Narrative Synthesis

Due to the methodological diversity of the included studies, relevant findings were narratively synthesized.

## Results

### Study Selection and Characteristics

**Figure 1:**
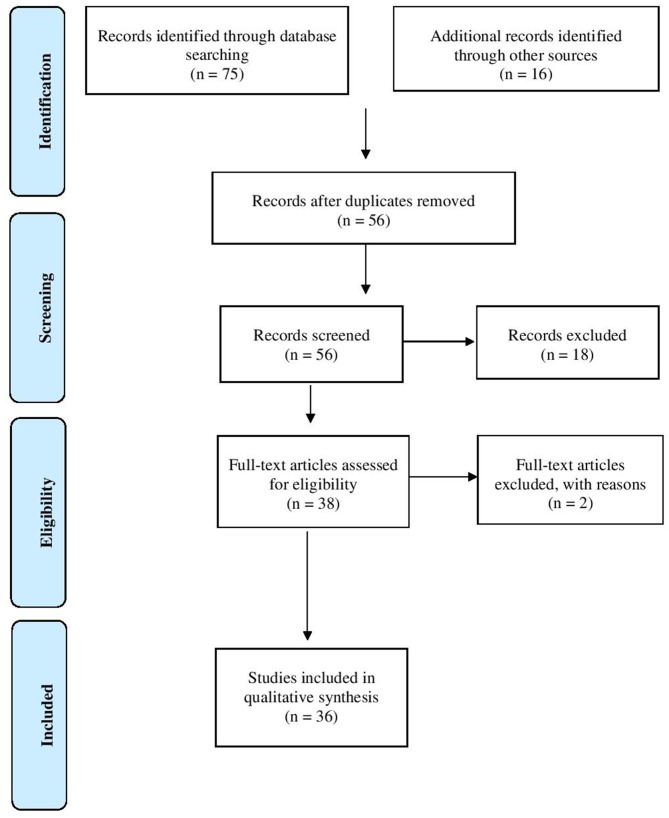
PRISMA flow-chart.

The systematic literature search yielded a total of 56 records following removal of duplicates. After screening of abstracts and closer examination of full-text papers, 20 articles were excluded as not relevant and the reasons for exclusion recorded (see Figure 1). Thus, the review included a total of 36 publications.

Characteristics of the included studies are presented in Supplementary Table 1 and summarized in Table 4. All papers were produced between 2006 and 2019. Most of the included studies were peer-reviewed journal articles (*N* = 33; 92%), produced in North America (*N* = 26; 72%), used quantitative methodologies (*N* = 34; 94%), cross-sectional designs (*N* = 24, 67%) and non-clinical samples (*N* = 29, 81%).

**Table 4:** Characteristics of included studies.

**Study focus category**	**Prevalence % (*N*)**	**Impact % (*N*)**	**Trajectory % (*N*)**	**Etiology % (*N*)**	**Treatment % (*N*)**	**Multiple % (*N*)**	**Total % (*N*)**
**Publication type**							
Peer-reviewed journal article	50% (1)	0% (0)	0% (0)	89% (16)	100% (4)	100% (12)	92% (33)
Unpublished dissertation	0% (0)	0% (0)	0% (0)	11% (2)	0% (0)	0% (0)	5% (2)
Conference abstract	50% (1)	0% (0)	0% (0)	0% (0)	0% (0)	0% (0)	3% (1)
**Country of origin**							
North America	100% (2)	0% (0)	0% (0)	78% (14)	25% (1)	75% (9)	72% (26)
Europe	0% (0)	0% (0)	0% (0)	17% (3)	75% (3)	25% (3)	25% (9)
Other	0% (0)	0% (0)	0% (0)	5% (1)	0% (0)	0% (0)	3% (1)
**Methodology**							
Quantitative	100% (2)	0% (0)	0% (0)	100% (18)	75% (3)	92% (11)	94% (34)
Qualitative	0% (0)	0% (0)	0% (0)	0% (0)	25% (1)	8% (1)	6% (2)
**Design**							
Cross-sectional	100% (2)	0% (0)	0% (0)	72% (13)	25% (1)	67% (8)	67% (24)
Longitudinal	0% (0)	0% (0)	0% (0)	28% (5)	75% (3)	33% (4)	33% (12)
**Sample**							
Non-clinical only	100% (2)	0% (0)	0% (0)	78% (14)	25% (1)	100% (12)	81% (29)
University students only	0% (0)	0% (0)	0% (0)	50% (9)	0% (0)	42% (5)	39% (14)
Females only	50% (1)	0% (0)	0% (0)	44% (8)	50% (2)	25% (3)	39% (14)

#### Study Focus

For each study focus category, descriptive statistics of publication type, country of origin, methodology, design, and sample are displayed in Table 4. Two studies focused solely on prevalence. No studies focused solely on trajectory or impact. Eighteen studies focused solely on etiology. Four studies focused solely on treatment. Twelve studies had multiple aims (e.g., etiology and prevalence). Study references are found in Supplementary Table 1.

#### Developmental Informedness

Percentage agreement between the two raters of developmental informedness was 88.9%. Following resolution of rater discrepancies, eight studies (22.2%) were rated as “strong” on developmental informedness. Twenty-two studies (61.1%) were rated as “moderate”. Six studies (16.7%) were rated as “weak.” Study references are found in Supplementary Table 1.

### Narrative Synthesis

The main findings of the included studies are narratively synthesized below, organized according to study focus. Study references are found in Supplementary Table 1.

#### Prevalence of Eating Disorders During Emerging Adulthood

Twelve studies reported the prevalence of EDs or their symptoms in EAs. Prevalence of binge-eating ranged between 4.4% in female university students and 13% in female Latina EAs (Goldschmidt et al., 2016; Pivarunas and Shomaker, 2016; Thurston et al., 2018; West et al., 2019). Regarding unhealthy weight control behaviors, one study reported that 26.4% of university student EAs engaged in unhealthy weight control behaviors (vomiting, fasting, excessive exercise, laxatives or diuretics) at least once per week (Hymowitz et al., 2017), whilst another found that 20.7% of female university students reported engaging in compensatory weight-control behaviors in the past year (Bankoff et al., 2013). The prevalence of restricted eating was not commonly investigated.

Regarding prevalence of probable diagnoses, one study found that 20.3% of EA university students scored above the clinical cut-off on the SCOFF, indicating probable ED (Hasselle et al., 2017). Another study found approximately comparable figures, reporting that 15.5% of female university students and 11.8% of males scored above the clinical cut-off on EAT-26 (Gonidakis et al., 2018). One study found that 11.5% of university student EAs met criteria for BED and 3.3% for night eating syndrome (Hymowitz et al., 2017), whilst another found a considerably higher figure of 31% of EAs meeting BED criteria (Patrick and Stahl, 2009). This small study (just 26 participants in the EA sample) was the only one to assess the prevalence of other EDs in EAs and found that 50% of male EAs and 31% of female EAs met criteria for AN, and 10% of males and 6% of females had BN (Patrick and Stahl, 2009). The prevalence of EDs in EAs did not differ from adolescent, midlife and later life comparison groups. However, these strikingly high figures may be an artifact of the small sample sizes in this study. Two studies examined the prevalence of EDs in EAs with T1 diabetes (T1D) (Bächle et al., 2015; Doyle et al., 2017). Both found prevalence of probable ED in females of ~30%, whilst rates for males were more variable: 9.5% (Bächle et al., 2015) vs. 18.2% (Doyle et al., 2017).

#### Trajectory of Eating Disorders From Adolescence to Emerging Adulthood

Two large longitudinal studies examined trajectories of ED symptoms over time. One study found mean level of drive for thinness decreased from adolescence to EA, whilst both body dissatisfaction and bulimia remained the same (Waszczuk et al., 2019). The other study provided a more detailed analysis of trajectory, and found that 8.2% of the population experienced overeating, binge-eating or BED in adolescence but these symptoms had remitted by EA, another 3.6% experienced overeating, binge-eating or BED in both adolescence and EA, whilst 7.2% were not experiencing these symptoms in adolescence but newly developed them during EA (Goldschmidt et al., 2016). Trajectories of other EDs (e.g., AN; BN) were not investigated.

#### Impact of Eating Disorders During Emerging Adulthood

One longitudinal study in a large, representative sample examined the impact of ED symptoms during EA on later development (Mason and Heron, 2016). Both objective over-eating and binge-eating (≥once per week) during EA were prospectively associated with a range of psychosocial functioning indices, including greater depressive symptoms, social isolation and sleep difficulties, lower perceived attractiveness and fewer close friends, in young adulthood. However, some of these relationships were no longer significant when controlling for depressive symptoms during EA. Additionally, the study failed to control for these psychosocial indices at baseline.

#### Etiology of Eating Disorders During Emerging Adulthood

##### Psychological factors

Twenty-two studies provided evidence of associations between psychological factors and EDs during EA. Cross-sectional studies indicated that effortful control, body appreciation or positivity, self-compassion, feelings of social safeness, resilience and positive perceptions of self were associated with lower levels of ED symptoms (Burt et al., 2015; Hymowitz et al., 2017; Thurston et al., 2018; Javier and Belgrave, 2019). Emotion regulation difficulties, negative emotionality, perceived stress, thin-ideal internalization or endorsement of societal messages about disordered eating, relationship avoidance and trait guilt were associated with higher levels of ED symptoms (Asberg and Wagaman, 2010; Bankoff et al., 2013; Lydecker et al., 2014; Burt et al., 2015; Hasselle et al., 2017; Hymowitz et al., 2017; Marta-Simões and Ferreira, 2018; Thurston et al., 2018; Javier and Belgrave, 2019). One case-control study found that those EAs with BN or BED had more early maladaptive schemas than EAs without these EDs, and that cognitions about eating and loss of control mediated the relationship between specific maladaptive schemata (e.g., schemata related to autonomy, disconnection, and vigilance) and food craving intensity. As this study did not include a comparison group of adolescents, it is not clear to what extent such schemata and cognitions are risk factors unique to EAs.

Findings were mixed regarding the relationship between depressive symptoms and ED symptoms. One cross-sectional study found no relationship between ED symptoms and depressive symptoms (Hasselle et al., 2017). Another study found that disordered eating was associated with both suicidality and depressive symptoms in a sample of predominantly female EAs (Mugoya et al., 2018) Another indicated that a range of ED symptoms were associated with depressive symptoms in female EAs, but only restrained eating was associated with depressive symptoms in male EAs (Rawana et al., 2016). Another study focused specifically on EAs with T1D, and found that female EAs with both ED and T1D had higher levels of depression than female EAs with T1D but without ED. However, there was no difference in depression levels in male EAs with T1D with and without EDs (Bächle et al., 2015). One large longitudinal study (reported in two publications) shed further light on the relationship between ED symptoms and depression (Goldschmidt et al., 2016; West et al., 2019). This study found that depressive symptoms in adolescence predicted ED in EA when controlling for ED during adolescence. Additional predictors of ED symptoms were low self-esteem and high body dissatisfaction (Goldschmidt et al., 2016; West et al., 2019).

One longitudinal study found that thought suppression during EA predicted ED symptoms in female university students three months later, when controlling for ED symptoms at the earlier time-point (Collins et al., 2014). Another study compared a T1D group and non-T1D group, and found that a self-esteem, mastery and optimism composite appeared to negatively predict EDs symptoms in EAs with T1D but not those without (Helgeson et al., 2014). This study did not include a comparison group of adolescents or adults; it is therefore not possible to determine the extent to which such psychological factors are uniquely relevant to EAs.

One cross-sectional study provided evidence that psychological characteristics posited to be distinctive to EA (identity exploration and experimentation/ sense of possibilities) are associated with ED symptoms (dieting; bulimia; oral control) during this life-stage (Gonidakis et al., 2018). Another study found that “quest orientation” (linked with religious identity development) was positively correlated with bulimia symptoms in 18-year old university students (Boyatzis and McConnell, 2006). However, no such relationship was found in third- and fourth-year university students or university graduates. Finally, a case-control study indicated that female EAs with AN scored higher on perceived personal uniqueness and self-consciousness, and reported higher psychological vulnerability, than both adolescents and EAs without AN (Fox et al., 2009). As this study did not include a comparison group of adolescents with AN, it is not clear to what extent such psychological characteristics are risk factors unique to EAs.

##### Social factors

Eight studies provided evidence of an association between social factors and EDs during EA. The studies using cross-sectional designs indicated that parenting style (degree of warmth and control), mother's ED symptoms, and experience of childhood physical abuse and polyvictimization were associated with EAs' ED symptoms (Lucas, 2010; Bankoff et al., 2013; Hasselle et al., 2017). A qualitative study found that peer support was experienced as a protective factor against disordered eating (Javier and Belgrave, 2019). Regarding longitudinal studies, a twin-study found that environmental factors contributed to both maintenance of ED symptoms from adolescence to EA and onset of symptoms during EA (Waszczuk et al., 2019). The twin-study methodology cannot provide information on the specifics of environmental factors involved. Another longitudinal study found that experience of rape or attempted rape was associated with an increased risk of disordered eating in female university students three months later, when controlling for disordered eating at the earlier time-point (Collins et al., 2014).

One longitudinal study explored whether progression to university, a social experience characteristic of EA, impacted ED symptoms in EAs with and without T1D (Palladino et al., 2013). The study found that ED symptoms (drive for thinness and bulimia) remained stable in those EAs who progressed to university. However, there were some changes amongst those who did not attend university; in the T1D group, drive for thinness increased, whilst the opposite pattern was evident in the non-T1D group (Palladino et al., 2013). Another longitudinal study explored ED symptoms in EAs with and without T1D and found that ED symptoms were predicted by conflict with friends in both groups. BN symptoms specifically were predicted by the interaction between parental support and conflict with friends, such that high levels of conflict with friends in the presence of low support from parents were associated with increased risk of BN symptoms. However, this study did not control for ED symptoms at baseline.

##### Genetic and other biological factors

Four studies have provided evidence of associations between genetic and other biological factors and EDs during EA. Studies using cross-sectional designs indicate that body mass index (BMI) is associated with ED symptoms, in both EAs with and without T1D (Doyle et al., 2017; Thurston et al., 2018). A longitudinal study found that respiratory sinus arrhythmia—a measure of parasympathetic nervous system activation—was associated with increased risk of ED symptoms six months later, independent of ED symptoms at baseline (Abaied et al., 2016). A twin-study reported that maintenance of ED symptoms from adolescence to EA was primarily due to the continued influence of stable genetic factors, whilst there was also evidence of the contribution of new genetic influences to changes in the course of symptoms between adolescence and EA (Waszczuk et al., 2019). A cross-sectional study focused specifically on EAs with T1D found that those EAs with probable ED had poorer metabolic control than EAs without probable ED (Doyle et al., 2017).

##### Interaction between psychological, social, and biological factors

Four studies have provided evidence of associations between psychological, social and biological interactions and EDs during EA. Self-perception was found to mediate the relationship between emotional abuse and ED symptoms (Hymowitz et al., 2017), whilst thought suppression moderated the effect of rape or attempted rape on ED symptoms three months later (Collins et al., 2014). Another longitudinal study reported that respiratory sinus arrythmia, parenting strategies and coping responses to stress interact to predict ED symptoms six months later (Abaied et al., 2016). One study found that whilst binge-eating was predicted by adolescent overweight/obesity in both high and low socioeconomic status (SES) groups, the strength of this relationship was greater in the high SES group than the low SES group (West et al., 2019). Additionally, adolescent body dissatisfaction and family weight-based teasing predicted binge-eating in the high SES group, but not the low. However, this study did not control for ED symptoms at or prior to baseline assessment.

### Treatment of Eating Disorders During Emerging Adulthood

Six studies investigated treatment of EDs during EA. Three studies focused on understanding the extent to which conventional adult service models and treatments work for EAs with EDs (Dimitropoulos et al., 2013; Weigel et al., 2014; Javier and Belgrave, 2019). One cross-sectional study compared duration of untreated ED in adolescents, EAs and adults with EDs, and found that EAs present to services with a longer duration of untreated illness than adolescents, although not as long as adults (Weigel et al., 2014). A qualitative study explored barriers and facilitators of ED treatment seeking specifically in Asian American EAs. It found that available resources and familial support were important facilitators of treatment-seeking, whilst stigma was a major barrier to accessing care (Javier and Belgrave, 2019). A qualitative study investigated clinicians' experiences of the transition between child and adolescent and adult ED services (Dimitropoulos et al., 2013). Many clinicians expressed the belief that the timing of transition from child to adult services should be determined by “readiness,” and not by age. Clinicians also identified interventions which they believed would improve the smoothness of transitions between services. Specifically, they highlighted the importance of educating parents about developmentally appropriate ways of supporting their child, and of fostering autonomy and independence in the patient.

Three studies investigated new approaches to intervention for EAs with EDs (Brown et al., 2018; McClelland et al., 2018; Koskina and Schmidt, 2019). A case-report described the treatment of an EA with recent onset AN. The patient was treated using the Maudsley Model of Anorexia Nervosa Treatment for Adults (MANTRA), but with enhanced focus on the identity-related aspects of this treatment. The patient showed significant sustained improved in both BMI and ED symptoms, and gave detailed positive feedback on her experience of treatment (Koskina and Schmidt, 2019). One study (reported in two publications) examined the feasibility, acceptability and effectiveness of First Episode Rapid Early Intervention for Eating Disorders (FREED) (Brown et al., 2018; McClelland et al., 2018). FREED is a service-model for specialist treatment of EAs with a recent onset ED and aims to both minimize wait-times for treatment and provide evidence-based interventions which have been adapted for EAs. Patients treated through FREED waited significantly less time from referral to assessment and treatment, and treatment uptake rates were significantly better, compared to previous practice within the service (Brown et al., 2018). Furthermore, FREED was associated with improvement in ED and co-morbid depression and anxiety symptoms over time, and BMI improvements in AN patients above treatment-as-usual (McClelland et al., 2018).

## Discussion

### Summary of Main Findings

The findings of the current systematic scoping review indicate that there has been some engagement with the concept of EA in the ED research literature. The majority of these studies originate from North America, have used quantitative methodologies, cross-sectional designs and non-clinical samples.

The studies included in this review have predominantly focused on understanding the etiology of EDs during EA, with some studies also assessing prevalence, trajectory, impact and treatment of EDs during EA. The majority of studies were informed by existing EA-focused developmental research to a moderate extent. The findings of the included studies are summarized below.

#### Prevalence of Eating Disorders During Emerging Adulthood

The present review found that ED symptoms are common amongst EAs; approximately a quarter of EAs engage in unhealthy weight control behaviors, whilst up to one-in-ten may engage in binge-eating, and between 11 and 20% have probable ED (Pivarunas and Shomaker, 2016; Hasselle et al., 2017; Hymowitz et al., 2017; Gonidakis et al., 2018). Such figures are on par with those found previously in university student samples (Eisenberg et al., 2011). The picture for prevalence of specific full-criteria EDs is less clear; existing research indicates that one-in-ten EAs meet criteria for BED (Hymowitz et al., 2017), but there is sparse data available for other diagnoses. The one study which compared prevalence rates amongst EAs with other age-groups found comparable rates across groups, although this study was deemed to be of poor quality, with particularly small sample sizes (Patrick and Stahl, 2009). There were some overall concerns about the methodological validity of existing prevalence-focused studies; most used small convenience samples of university students and self-report measure of ED symptoms, which generate estimates of probable EDs at best.

#### Trajectories of Eating Disorders During Emerging Adulthood

Studies included in the present review indicate that trajectories into ED during EA are diverse, and those experiencing ED symptoms during EA are not necessarily the same people who experienced ED symptoms during adolescence (Goldschmidt et al., 2016).

#### Impact of Eating Disorders During Emerging Adulthood

This review found that sparse research has investigated the long-term impact of ED during EA; however, there is evidence from one study that binge-eating during EA impacts a broad range of psychosocial outcomes in later adulthood (Mason and Heron, 2016). This is consistent with existing literature, which shows that ED has a lasting impact on psychosocial functioning (Maxwell et al., 2011). However, it appears that such effects are entangled with co-occurring depressive symptoms. Furthermore, given inadequate control for potential confounders, the extent to which such psychosocial outcomes are independent from pre-existing psychosocial difficulties remains unclear.

#### Etiology of Eating Disorders During Emerging Adulthood

The present review found that a broad range of psychological, social and biological factors are associated with EDs during EA. These factors are not present in isolation, but rather appear to interact with other variables (Collins et al., 2014; Abaied et al., 2016; Hymowitz et al., 2017; West et al., 2019). However, most of these studies are cross-sectional in design, limiting the inferences that can be made about causality. Additionally, very few of these studies included relevant comparison groups (e.g., adolescents; young adults), limiting the extent to which such factors can be said to be particularly relevant to EAs. Indeed, these findings are broadly consistent with existing research regarding risk factors for EDs in adolescence and adulthood and suggest there is some shared etiology of EDs during both EA and other life-stages (Jacobi et al., 2004; Allen et al., 2013a,b, 2014). However, there is also some tentative evidence of relationships between the specific psychosocial characteristics of EA (e.g., identity exploration) and ED etiology (Boyatzis and McConnell, 2006; Gonidakis et al., 2018). Furthermore, there is evidence that new genetic influences which influence ED come online during EA (Waszczuk et al., 2019). The transition to university was found to have no impact on ED symptoms (Palladino et al., 2013). This is perhaps surprising, given existing qualitative research which has found that ED symptoms tend to worsen during this transition (Goldschen et al., 2019). However, there are some concerns regarding the included study's methodological validity (e.g., lack of comprehensive assessment of ED symptoms), and its findings should be regarded with caution.

#### Treatment of Eating Disorders During Emerging Adulthood

This review found that studies focusing specifically on treatment of EDs during EA have identified several issues with existing ED adult services for EAs (Dimitropoulos et al., 2013; Weigel et al., 2014; Javier and Belgrave, 2019). EAs present to ED services later than adolescents, and stigma, lack of resources and lack of familial support may be key barriers to help-seeking in EA populations (Weigel et al., 2014; Javier and Belgrave, 2019). These findings are broadly consistent with current understandings of delayed help-seeking for other mental health problems in EA populations (Spence et al., 2016). Facilitating the transition from child and adolescent services to adult services appears to be an issue of particular concern for ED clinicians (Dimitropoulos et al., 2013). Clinicians identified the importance of both parental support and of autonomy development in facilitating smoother transitions (Dimitropoulos et al., 2013). This echoes findings regarding clinicians' views of transitions between adolescent and adult mental health services more broadly, emphasizing that these concerns are not unique to ED populations and integration of research across diagnoses is likely to be useful (Hovish et al., 2012). Despite the apparent need the present review found no studies evaluating potential models of transition between ED services. However, this review found that evaluations of adult ED services and treatments adapted to the needs of EAs have produced promising results. Specifically, treatment within the FREED model was associated with significant improvement in ED and co-morbid depression and anxiety symptoms over time, alongside larger BMI improvement in AN patients compared to treatment as usual (Brown et al., 2018; McClelland et al., 2018; Koskina and Schmidt, 2019). Although this research does not identify mechanism of effect, key aspects of this service model include rapid access to care, flexible caregiver involvement and a focus on identity development and management of transitions.

### Limitations

Existing studies have for the most part focused on understanding the etiology of ED onset and maintenance during EA. There are few studies delineating incidence or prevalence of BN or AN during EA. This is not surprising, as epidemiological studies typically assess ED incidence or prevalence in age-groups that do not align well with the boundaries of EA (e.g., 15–19 years; 20–24 years; Micali et al., 2013). Given that the boundaries of EA more closely align service provision demarcations (under 18 years vs. over 18 years), assessing epidemiology in the 18–25-year age group specifically may prove more useful for planning service provision.

Existing etiologically focused studies have predominantly focused on uncovering psychological or social factors involved in EDs during EA, with a comparative lack of focus on biological factors. This reticence within the biological field to explore EDs within the context of EA may reflect a perception that EA is a psychosocial construct. However, existing evidence that EA is associated with distinct patterns of biological development suggest that there is much to be gained from the evaluation of biological mechanisms within this population. Furthermore, etiological studies have also tended to examine either psychological, social or biological factors, rather than take an interdisciplinary approach. EA's distinctive biological, psychological and social characteristics should not be considered in isolation, but instead are likely to be closely intertwined. Understanding EDs within the context of EA will require consideration of all levels, and how they interact.

Additionally, there are a number of methodological concerns regarding existing studies. The majority of etiologically focused studies have been cross-sectional in design. Cross-sectional studies are less well-suited to understanding etiology than longitudinal designs, and the findings of these studies should be interpreted carefully. Furthermore, studies have not tended to include comparison groups of adolescents and/or young adults, so it is not clear to what extent the explored factors are relevant to EAs only, or also to other populations.

Many studies did not include developmental indices and tended to examine variables that are of interest in ED populations generally. It is important to research these variables, as they may have a differential effect in EA populations compared to adolescents or adults. However, a truly developmental approach to understanding EDs during EA does not merely involve studying already-evidenced factors in EA populations (Cicchetti and Rogosch, 2002). Rather, EDs might be usefully conceptualized in terms of how they relate to the normative developmental tasks of EA (Cicchetti and Rogosch, 2002). For instance, understanding of normative EA brain development could provide valuable insights into some of the mechanisms that eventuate in or maintain ED (Taber-Thomas and Pérez-Edgar, 2015). Indeed, MRI studies have revealed that EDs are associated with alterations in brain structures and functions that are known to be maturing during EA (Frank, 2015). It may be that deviation from the processes underlying normal brain development might contribute to EDs. Similarly, in keeping with long-standing theorizing regarding connections between identity and EDs, divergence from normative identity development might also contribute to ED etiology (Bruch, 1981; Verschueren et al., 2017a; Oldershaw et al., 2019). Unraveling these connections has the potential to greatly enhance our understanding of EDs during EA.

Quantitative methodologies predominate in existing research, with a comparative lack of qualitative research. Whilst quantitative methodologies are appropriate when research questions are unambiguous, and when variables can be isolated and defined, qualitative research is useful for understanding more complex phenomena (Hammarberg et al., 2016). Given that EA is one such complex phenomenon, it is likely that qualitative methodologies have much to contribute to our understanding of EDs as they occur during this life-stage. Qualitative methodologies are also particularly well-suited to questions related to experience and meaning and could be well-placed to explore the EAs' own views on how their ED treatment needs would be best met. Indeed, qualitative and quantitative methodologies should not be considered as mutually exclusive, but instead can often be used to complement each other. For instance, qualitative research might be used to generate hypotheses and quantitative studies used to test these hypotheses at a population-level.

Finally, much of the existing research has been conducted in university student samples. Little is known about the extent to which existing findings in EA university students can be generalized to the population at large, and non-university attending 18 to 25-year-olds. Additionally, there is a clear preponderance of EA samples in Western cultural contexts, with most research conducted in the United States. As with non-university attending EAs, it is important to explore the extent to which patterns also apply to EAs in non-Western countries.

### Future Research

The current review makes clear that many unanswered questions remain regarding EDs during EA. In particular, future studies should aim to identify the prevalence and incidence rate of EDs in EAs, compared to both adolescents and young adults. Additionally, research should aim to elucidate what unique and overlapping risk factors exist for different EDs during EA, compared to both adolescence and adulthood. There are also many questions to be addressed regarding treatment of EDs during EA. For instance, future research might usefully explore whether ED services should be trans-age, or whether EAs are best served in young peoples' services. Additionally, research should aim to identify what developmental changes—if any—need to be made to standard evidence-based treatments for EDs to best accommodate the needs of EAs. Future research should endeavor to answer these questions whilst paying careful attention to methodological validity and avoiding the pitfalls of existing studies, as identified in this paper's limitations section.

### Clinical Implications

Arising from the findings of this review, several tentative suggestions can be made as to how ED services and the interventions they provide might be tailored to EAs' needs.

#### Support resolution of normative developmental tasks

As indicated by the findings of the present review, ED during EA is likely to hinder resolution of developmental tasks, and failure to resolve these in a timely fashion has the potential to derail psychosocial development (Mason and Heron, 2016). Normative resolution of developmental tasks, where possible, may limit long-term impairment associated with EDs. Reviewed studies found that difficulties with developmental tasks may also precipitate or maintain an ED, and attempts to work toward resolution of these tasks may be therapeutic in and of themselves (Gonidakis et al., 2018; Koskina and Schmidt, 2019). It might therefore be recommended that service providers and clinicians acknowledge that EAs are engaged in developmental tasks and aim to support them with the resolution of these tasks in so far as is possible. Indeed, one reviewed study found that there is appetite amongst clinicians to focus on developing and practicing the skills required for independent living amongst EA patient populations (Dimitropoulos et al., 2013). Skills might be related to self-management of illness (e.g., meal planning and preparation, medication management), but also more general (e.g., time-management, budgeting). Given the findings of studies included in the present review, offering support related to identity development may be a particularly fruitful avenue (Boyatzis and McConnell, 2006; Gonidakis et al., 2018; Koskina and Schmidt, 2019). This may include offering pre-existing psychological interventions that include identity-focused interventions and have been found to be effective in EA populations (e.g., MANTRA; Schmidt et al., 2015; Koskina and Schmidt, 2019) or more practical support around career development, for instance. Social media is a ubiquitous vehicle for identity exploration amongst EAs, and support and assessment around this may also be useful. Indeed, social media-focused support is a component of the FREED service model, which has been found to be associated with clinical improvement above treatment-as-usual (Brown et al., 2018; McClelland et al., 2018).

#### Balance self-management with caregiver-support

The developmental literature on EA indicates that EAs have an in-between level of autonomy and decision-making capabilities (Arnett and Mitra, 2018; Steinberg et al., 2018). One study included in this review indicates an awareness amongst clinicians that readiness to take responsibility for ED-related healthcare and treatment does not always align with turning eighteen (Dimitropoulos et al., 2013). At best, the demands adult services place on EAs regarding self-management of illness may be off-putting and developmentally challenging; at worst they may contribute to treatment disengagement and symptom deterioration. Conversely, the heavy emphasis on caregiver support characteristic of adolescent services is also likely to be off-putting for EAs. It might therefore be recommended that service providers and clinicians aim to strike a balance between incorporating caregiver support and patient independence when treating EAs (Winston et al., 2012; Garland et al., 2018). There is some evidence for the effectiveness of these types of approaches; the FREED service-model emphasizes patient-led caregiver-inclusion (e.g., giving the option to bring caregiver(s) to their assessment appointment) and has been shown to be associated with clinical improvement above treatment-as-usual (Brown et al., 2018; McClelland et al., 2018). Family therapy might also be offered, but ideally adapted to the needs of EAs. One existing model—family-based therapy for transition-aged youth (TAY-FBT)—is not explicitly focused on EA, and thus has not been included in this systematic scoping review. However, its underpinning framework of “transition-aged youth” does share much conceptual overlap with EA. It aims to strike a balance between collaboration between the young person and their family, whilst maintaining developmentally appropriate autonomy. An open trial of TAY-FBT has recently shown promising outcomes for individuals with AN (Dimitropoulos et al., 2018). Another pilot study of FBT for young adults, which had a similar collaborative approach, has also shown promise (Chen et al., 2016).

This review highlights the current lack of research regarding the impact of transition to university on ED during EA. However, this transition is likely to be a particularly key time to reassess the self-management/caregiver support balance, as it is a major step up in terms of independence. EAs are often living away from parents for the first time, in an academic environment that is associated with a less structured timetable. Such normative developmental challenges are likely to be detrimental for someone already experiencing an ED or vulnerable to developing one. It might be tentatively suggested—pending insights provided by further research—that clinicians should aim to support decision-making related to starting or returning to university, and work with EAs to carefully consider potential benefits vs. harms of continuing studies. Helpful guidance exists for fitness to study for students with severe EDs, and this should be used collaboratively by clinicians to facilitate optimum decision-making and planning (Higher Education Occupational Physicians/Higher Educational Occupational Practitioners, 2018). For those who do decide to return to university, there is some evidence included in this review that offering psychoeducational groups which focus on developing the skills of independent living (e.g., time-management, work/life balance, budgeting, meal planning, managing medications) may be useful (Dimitropoulos et al., 2013; Brown et al., 2018; McClelland et al., 2018).

#### Facilitate smooth care transitions

The developmental literature indicates that EAs' lives are characterized by instability in many life domains (e.g., occupation, residence relationships) (Arnett, 2000). This review highlights that further research is needed regarding the impact of such instability on EAs experiencing EDs. However, frequent residence changes are likely to be of particular relevance to ED service and treatment provision, given that conventional services tend to assume that the patient will continue to be able to access care from their initial residence through their prescribed course of treatment, and the strong therapeutic relationships that develop with continuity of contact are understood to be integral to successful outcomes (Macdonald et al., 2019). There is evidence that care transitions are often poorly planned and far from seamless and can be detrimental to patient outcomes (Dimitropoulos et al., 2013; Parliamentary and Heath Service Ombudsman, 2017). Furthermore, a study included in this review indicates that many EAs do not yet have the skills for independent living and decision-making and may struggle with navigating complex healthcare systems and setting up support in their new location (Dimitropoulos et al., 2013). Arising from this, service providers and clinicians should be aware that EAs are likely to encounter a care transition and aim to make these transitions as seamless as is possible. This might include offering timely practical support around setting up help in the EA's new location, information-giving about how to register with a new GP, and advance consideration of what ongoing support may be needed. It might be tentatively suggested that periods of parallel care, whereby the EA has two ED teams, may be helpful and appropriate. This is particularly applicable when the EA has gone away to university and will return to their hometown for lengthy breaks between university terms, as it will allow them to receive treatment both during term-time and during the holidays.

#### Embrace individuality

The developmental literature indicates that EA is a heterogeneous life-stage, and no two EAs' social context, level of development and needs are likely to be the same (Arnett, 2000). As indicated by one study in the present review, this has implications for ED services; one 18 year old might be ready to self-manage their ED, whilst another might not be (Dimitropoulos et al., 2013). Additionally, the findings of one reviewed study imply that some EAs are coming to ED services for the first time, whilst others will have been experiencing ED since adolescence (Goldschmidt et al., 2016). “One size fits all” services and treatment models are unlikely to suit (Dimitropoulos et al., 2013). It might be suggested that service providers and clinicians should be aware of the heterogeneity of this life-stage and aim to tailor treatments to the unique needs of each EA. Services should aim for case-by-case assessment of developmental context and needs and be prepared to adjust treatment accordingly. For instance, given the variation in their living situations, EAs may have access to a range of possible support people (e.g., partners; friends; parents; siblings; coaches; university tutors), flexibility around who is considered a “caregiver” may be useful. It is important that patients be reassessed on an ongoing basis, as needs will change as stage of development, stage of illness and context changes.

#### Provide hope for the future

The developmental literature indicates that EAs tend to be optimistic about their future and feel that change is possible (Arnett, 2000). Regarding EDs, this optimism is not necessarily misplaced; there is evidence that it is possible to achieve total recovery—including reversal of ED-related brain changes and impact on fertility—if treatment occurs quickly (Bulik et al., 1999; Crow et al., 2002; Frank, 2015). Indeed, studies included in the present review demonstrate that significant clinical improvement is possible with rapid access to evidence-based treatment (Brown et al., 2018; McClelland et al., 2018). Conventional adult ED services do often promote messages of recovery, whilst still acknowledging and accepting that some patients will not improve, and quality of life may remain impaired. However, EAs may benefit from a greater, explicit emphasis on hope for full sustained recovery. Indeed, one study found that young people with experiences of ED treatment expressed dissatisfaction at the possibility of recovery not being discussed (Mitrofan et al., 2019). It might be tentatively suggested that service providers and clinicians should cater to EAs' sense of optimism and create services that emphasize that full recovery is an achievable and desirable goal. For instance, services might consider employing peer workers or incorporating recovery stories in their written materials. Psychoeducation which emphasize that EAs' brains are highly plastic, and it is possible to recoup ED-related brain changes, might also be useful.

## Conclusion

Existing research indicates that the concept of EA brings a unique and valuable perspective to our understanding of EDs during the transition to adulthood. There is evidence that EA's specific psychosocial characteristics may contribute to ED etiology, and ED services for EAs should implement adaptations which exploit the opportunities and overcome the challenges of this developmental stage. Despite this, the concept of EA remains underused in ED research. Future engagement with the developmental literature by both researchers and clinicians may be key to understanding and treating EDs across the lifespan.

## Author Contributions

RP and US designed the review. RP and KR jointly conducted the systematic searches and completed data extraction. RP wrote the manuscript, with critical revisions from US, KA, and KR. All authors read and approved the final manuscript prior to submission.

### Conflict of Interest

The authors declare that the research was conducted in the absence of any commercial or financial relationships that could be construed as a potential conflict of interest.
