# Supplementary material for: Eating Disorders During Emerging Adulthood: A Systematic Scoping Review
Source: Front Psychol. 2020 Jan 31;10:3062. doi: 10.3389/fpsyg.2019.03062 (PMC7005676; doi:10.3389/fpsyg.2019.03062)
Supplement: Supplementary file 1 [file Table_1.docx]

Supplementary Table 1. Characteristics of included studies

| Study/country | | Publication type | | Objective of interest | Developmentally Informed | Methodology | Design | Sample | | N | Mean ± SD age (years) | Findings |  |
| --- | --- | --- | --- | --- | --- | --- | --- | --- | --- | --- | --- | --- | --- |
| Prevalence |  |  |  | |  |  |  |  | |  |  |  |  |
| Bachle et al. ^a^ Germany | 2015 | Peer-reviewed journal article | Prevalence of EDs in male + female EAs with early-onset T1D | | Moderate | Quantitative | Cross-sectional | Female  T1D  Occupation NR Race/Ethnicity NR  Male  T1D  Occupation NR Race/Ethnicity NR | | 126    85 | 19.4 ± 1.0  19.3 ± 0.9 | Probable ED (≥ 2 on SCOFF): 9.5% of males; 30.2% of females; males < females |  |
| Bankoff et al. ^a^ USA | 2013 | Peer-reviewed journal article | Prevalence of compensatory weight-control behaviour in EAs | | Moderate | Quantitative | Cross-sectional | Female  NC  Uni students 56% White 27% Asian 17% Other | | 759 | 19.2 ± 2.0 | Compensatory weight-control behaviour (≥ 1 in past year): 20.7% |  |
| Doyle et al. ^a^ USA | 2017 | Peer-reviewed journal article | Prevalence of disordered eating in male + female EAs with T1D | | Weak | Quantitative | Cross-sectional | Female T1D  Occupation NR  Male T1D  Occupation NR  Race/Ethnicity (TS): 83% White | | 27  33 | 20.6 ± 2.5  21.2 ± 2.6 | Disordered eating (>20 on DEPS-R): 29.6% of females; 18.2% of males; males = females |  |
| Gonidakis et al. ^a^ Greece | 2018 | Peer-reviewed journal article | Prevalence of “at-risk” ED status in male + female EAs | | Strong | Quantitative | Cross-sectional | Female  NC Uni students Race/Ethnicity NR  Male  NC  Uni students Race/Ethnicity NR | | 252  85 | 20.8 ± NR  21.3 ± NR | “At risk” for ED (≥ 20 on EAT-26): 15.5% of females; 11.8% of males; males = females |  |
| Hasselle et al. ^a^ USA | 2017 | Peer-reviewed journal article | Prevalence of EDs in EAs | | Moderate | Quantitative | Cross-sectional | 72% female  NC  Uni students 66% White 17% Black 17% Other | | 288 | 19.2 ± 1.4 | Probable ED (≥ 2 on SCOFF): 20.3% |  |
| Hymowitz et al. ^a^ USA | 2017 | Peer-reviewed journal article | Prevalence of BED / NES / unhealthy weight-control behaviours in EAs | | Moderate | Quantitative | Cross-sectional | 60% female  NC Uni students 46% White 31% Asian 33% Other | | 598 | 19.5 ± 1.5 | BED: 11.5%  NES: 3.3%  Unhealthy weight-control behaviour (≥ 1 per week): 26.4% |  |
| Mason & Heron ^a^  USA | 2016 | Peer-reviewed journal article | Prevalence of objective over-eating / loss of control eating in EAs | | Moderate | Quantitative | Longitudinal | 53% female  NC  Occupation NR Race/Ethnicity NR | | 12288 | NR | Objective over-eating (≥ 1 in past week): 5.9%  Loss of control eating (≥ 1 in past week): 2.1% |  |
| Pivarunas & Shomaker ^a^  USA | 2016 | Conference abstract | Prevalence of ED symptoms in Latina + White American EAs | | Moderate | Quantitative | Cross-sectional | 100% Latina  Female  NC  Occupation NR  100% White  Female  NC  Occupation NR | | 142  510 | 18.7 ± 1.1 (TS) | Binge-eating (frequency NR): 8% of White; 13% of Latina; Latina > White  Dieting + food preoccupation (frequency NR): (% NR); Latina = White  Eating restraint (frequency NR): (% NR); Latina > White |  |
| Patrick & Stahl USA | 2009 | Peer-reviewed journal article | Prevalence of ED symptoms in four age groups | | Moderate | Quantitative | Cross-sectional | Late adols NC 68% female  EAs NC 68% female  Midlife NC 68% female  Later life NC 68% female  Race/ Ethnicity (TS): 94% White 5% Black 1% Other | 43  26  27  29 | | 18.0 ± 0.0  21.8 ± 1.1  44.6 ± 4.7  59.7 ± 10.9 | Prevalence of AN in female EAs: 31.3%  AN in male EAs: 50.0%  BN in female EAs: 6.3%  BN in male EAs: 10.0%  Bingeing in female EAs: 32.0%  Bingeineg in male EAs: 30.0%  No differences between the groups in % with putative ED (AN; BN; bingeing) diagnoses |  |
|  |  |  |  | |  |  |  |  | |  |  |  | |
| Thurston et al. ^a^ USA | 2018 | Peer-reviewed journal article | Prevalence of binge-eating in EAs | | Moderate | Quantitative | Cross-sectional | Female  NC Uni students 49% White 27% Black 34% Other | | 297 | 19.2 ± 1.5 | Moderate bingeing (18-26 on BES): 12.5%  Severe bingeing (>27 on BES): 4.4% | |
| West et al. ^a^  USA | 2019 | Peer-reviewed journal article | Prevalence of binge-eating in low SES + high SES EAs | | Weak | Quantitative | Longitudinal | Low SES  NC  55% female Occupation NR 50% White 27% Asian 33% Other  High SES  NC  50% female Occupation NR 81% White 10% Asian 9% Other | | 1187      992 | NR      NR | Binge-eating (≥ 1 in past year): Low SES: 6.3%; High SES: 4.9%; Low SES = high SES | |
| Trajectory |  |  |  | |  |  |  |  | |  |  |  | |
| Goldschmidt et al. ^a^ USA | 2016 | Peer-reviewed journal article | Stability of overeating / binge-eating / BED between adolescence + EAs | | Weak | Quantitative | Longitudinal | 57% female NC Occupation NR 67% White | | 1827 | NR | Overeating (≥ 1 in past year), binge-eating (≥ 1 in past year) or BED:  No symptoms adolescence + EA: 81%  Symptoms remit adolescence to EA: 8.2%  Symptoms maintained adolescence to EA: 3.6%  Symptoms developed adolescence to EA: 7.2% | |
| Waszczuk et al. ^a^ Norway | 2019 | Peer-reviewed journal article | Change in ED symptoms between adolescence + EA | | Moderate | Quantitative | Longitudinal | 57% female NC  Occupation NR Race/Ethnicity NR | | 1453 | 19.6 ± 2.0 | Drive for thinness: adolescence < EA  Body dissatisfaction: adolescence < EA  Bulimia: adolescence = EA  Longitudinal cross-symptom correlations among ED symptoms were moderate to high, with drive for thinness and body dissatisfaction being the highest | |
| Impact |  |  |  | |  |  |  |  | |  |  |  | |
| Mason & Heron ^a^ USA | 2016 | Peer-reviewed journal article | Impact of objective over-eating / loss of control eating during EA on psychosocial adjustment in young adulthood | | Moderate | Quantitative | Longitudinal | 53% female NC  Occupation NR Race/Ethnicity NR | | 12288 | NR | Objective over-eating (≥ 1 in past week) during EA associated with:  +social isolation  -perceived attractiveness  in young adulthood.  Loss of control eating (≥ 1 in past week) during EA associated with:  +depressive symptoms  +social isolation  +sleep difficulty  -close friends  in young adulthood. | |
| Etiology |  |  |  | |  |  |  |  | |  |  |  | |
| Abaied et al. USA | 2016 | Peer-reviewed journal article | Impact of RSA, coping responses + parent psychological control on ED symptoms in EA | | Moderate | Quantitative | Longitudinal | 85% female NC Uni students 85% White 11% NR 4% Other | | 66 | 19.4 ± 0.8 | ED symptoms predicted by interaction between RSA, parent psychological control + coping responses | |
| Asberg & Wagaman USA | 2010 | Peer-reviewed journal article | Relationship between body dissatisfaction, perceived stress, social support, emotion regulation + ED symptoms in EAs | | Moderate | Quantitative | Cross-sectional | 70% female NC  Uni students 88% White | | 95 | 18.9 ± 1.5 | ED cognitions and behaviours positively associated with perceived stress  ED cognitions associated with less effective use of emotion reduction as a regulation strategy | |
| Bachle et al. ^a^ Germany | 2015 | Peer-reviewed journal article | Relationship between, metabolic control, depressive and ED symptoms in EAs with early-onset T1D | | Moderate | Quantitative | Cross-sectional | Probable ED (≥ 2 on SCOFF) 100% female  T1D Occupation NR Race/Ethnicity NR  No ED 100% female T1D Occupation NR Race/Ethnicity NR  Probable ED (≥ 2 on SCOFF)  100% male  T1D Occupation NR Race/Ethnicity NR  No ED  100% male  T1D Occupation NR Race/Ethnicity NR | | 211 (TS) | 19.4 ± 1.0 (TS) | Depressive symptoms: ED female group > no ED female group; ED male group = no ED male group  All females: ED symptoms associated with depressive symptoms  All males: Trend-level correlation between ED symptoms and depressive symptoms  No association between ED symptoms and metabolic control (HbA1c) in either group | |
| Bankoff et al. ^a^  USA | 2013 | Peer-reviewed journal article | Relationship between childhood abuse + adult attachment styles on compensatory weight-control behaviours in EA | | Moderate | Quantitative | Cross-sectional | Female NC Uni students 56% White 27% Asian 17% Other | | 759 | 19.2 ± 2.0 | Compensatory weight-control behaviours associated with:  + Relationship avoidance  + Global psychosocial functioning  + Childhood abuse (trend-level only)  Interaction between childhood abuse and relationship avoidance  Not associated with perceived power in sexual relationships | |
| Boyatzis & McConnell USA | 2006 | Peer-reviewed journal article | Relationship between Quest orientation and ED symptoms in EAs | | Strong | Quantitative | Cross-sectional | 1^st^ /2^nd^ year uni Female  NC  3^rd^ / 4^th^ year uni Female  NC  Uni graduates Females  NC  Majority White (TS; frequency NR) | | 57  43      51 | 18.6 ± 0.7  20.9 ± 0.5    25.3 ± 1.1 | 1^st^ /2^nd^ year uni: ED symptoms (bulimia + body dissatisfaction) associated with higher Quest scores  3^rd^ / 4^th^ year uni: ED symptoms not associated with Quest scores  Uni graduates: ED symptoms not associated with Quest scores | |
| Burt et al. USA | 2015 | Peer-reviewed journal article | Relationship between negative emotionality, effortful control and ED symptoms in EAs | | Weak | Quantitative | Cross-sectional | 52% Female NC Uni students 46% White 31% Black 23% Other | | 160 | 19.7 ± 1.7 | ED symptoms associated with:  - Effortful control  Interaction between effortful control + negative emotionality | |
| Collins et al. USA | 2014 | Peer-reviewed journal article | Impact of recent rape / attempted rape + thought suppression on ED symptoms in EAs | | Moderate | Quantitative | Longitudinal | 100% Female  NC Uni students 74% White  10% Black 16% Other | | 319 | 18.0 ± 0.4 | ED symptoms predicted by:  Rape/attempted rape  Thought suppression  Interaction between thought suppression + rape/attempted rape | |
| Doyle et al. ^a^  USA | 2017 | Peer-reviewed journal article | Relationship between T1D clinical characteristics and ED | | Weak | Quantitative | Cross-sectional | Probable ED 57% Female T1D Occupation NR  No ED 41% Female T1D  Occupation NR  83% White (TS) | | 14  46 | 20.1 ± 2.5  21.0 ± 2.6 | Metabolic control (HbA1c levels): ED group > non-ED group  No other group differences (sex; age; diabetes duration; age at diagnosis; treatment choice)  Whole-group associations:  Disordered eating associated with poorer metabolic control (higher HbA1c levels) and higher BMI | |
| Fox et al. UK | 2009 | Peer-reviewed journal article | Compare egocentric beliefs in EAs with EDs + EAs without EDs + adolescents without EDs | | Strong | Quantitative | Cross-sectional | AN  EAs  100% Female Occupation NR 100% White  NC EAs 100% Female  Occupation NR 100% White  NC Adols  100% female  Occupation NR Race/Ethnicity NR | | 31    26    71 | 22.9 ± 2.3    22.8 ± 3.5    16.9 ± 0.3 | Personal uniqueness: AN group > EA HC group + Adolescent HC group  Public Self-Consciousness: AN group > EA NC group + Adol. NC group  Psychological invulnerability: AN group < EA NC group + Adol. NC group  Within-group associations (AN):  Eating concern associated with:  + public self-consciousness  + danger invulnerability  +doubts about being understood | |
| Goldschmidt et al. ^a^ USA | 2016 | Peer-reviewed journal article | Impact of adolescent BMI, depressive symptoms, body dissatisfaction + self-esteem on overeating, binge-eating and BED during EA | | Weak | Quantitative | Longitudinal | 100% female  NC  Occupation NR  100% male  NC  Occupation NR  67% White (TS) | | 1040  787 | NR  NR | In female group, ED symptoms predicted by:  +Depressive symptoms  -Body satisfaction  -Self-esteem  In male group, ED symptoms predicted by:  +BMI  +Depressive symptoms  -Body satisfaction | |
| Gonidakis et al. ^a^ Greece | 2018 | Peer-reviewed journal article | Relationship between characteristics of EA + ED symptoms in male and female EAs | | Strong | Quantitative | Cross-sectional | 100% female  NC Uni students Race/Ethnicity NR  100% male  NC Uni students Race/Ethnicity NR | | 252  85  85 | 20.8 ± NR  21.3 ± NR | In female group, ED symptoms associated with:  Identity exploration  Experimentation / possibilities  Negativity / instability  Higher identity exploration associated with higher probability of ED “at risk status” in females (≥ 20 on EAT-26)  In male group, ED symptoms not associated with EA characteristics.  Lower identity exploration associated with higher probability of ED “at risk status” in males (≥ 20 on EAT-26) | |
| Hasselle et al. ^a^  USA | 2017 | Peer-reviewed journal article | Relationship between polyvictimisation + ED symptoms in male + female EAs | | Moderate | Quantitative | Cross-sectional | 72% female  NC Uni students 66% White 17% Black 17% Other | | 288 | 19.2 ± 1.4 | ED symptoms associated with:  +Childhood polyvictimisation  +Emotion regulation difficulties  No association between ED symptoms + PTSD symptoms or depressive symptoms | |
| Helgeson et al. USA | 2014a | Peer-reviewed journal article | Impact of self-esteem, mastery + optimism on ED symptoms in EAs with + without T1D | | Moderate | Quantitative | Longitudinal | T1D 53% female 75% uni students 93% White  NC 53% female 74% uni students 93% White | | 118  122 | 18.2 ± 0.4  18.0 ± 0.5 | In T1D group, ED symptoms predicted by self-esteem, mastery + optimism composite  In NC group, self-esteem, mastery + optimism composite did not predict ED symptoms | |
| Helgeson et al. USA | 2014b | Peer-reviewed journal article | Impact of parent + peer relationships on ED symptoms in EAs with + without T1D | | Strong | Quantitative | Longitudinal | T1D  53% female  75% uni students 92% White  NC 54% female  75% uni students 93% White | | 117  122 | 18.2 ± 0.4  18.0 ± 0.5 | In both T1D + NC group:  ED symptoms (bulimia + drive for thinness; EDI) predicted by friend conflict  Bulimia symptoms predicted by low parent support x high peer conflict | |
| Hymowitz et al. USA | 2017 | Peer-reviewed journal article | Impact of emotional abuse on disordered eating through negative self-perception in EAs | | Moderate | Quantitative | Cross-sectional | 60% female  NC  Uni students 46% White 31% Asian 23% Other | | 598 | 19.5 ± 1.5 | Significant positive associations between emotional abuse and disordered eating, and disordered eating and self-perception + BMI  Indirect effect of emotional abuse on disordered eating through self-perception  Emotional abuse has moderate to high-level specificity as a predictor of BED and NES | |
| Javier & Belgrave USA | 2018 | Peer-reviewed journal article | Barriers + facilitators of disordered eating in Asian American EAs | | Moderate | Qualitative | Cross-sectional | 100% female At risk for ED Occupation NR  100% Asian | | 26 | 19.3 ± 0.8 | Facilitators of disordered eating:  Endorsement of messages about disordered eating  Self-related challenges  Barriers to disordered eating:  Body positivity  Peer support | |
| King USA | 2012 | Unpublished dissertation | Relationship between strength of religious faith, value endorsements+ ED symptoms in EAs | | Moderate | Quantitative | Cross-sectional | 77% female NC  Uni students 90% White | | 99 | 19.1 ± 1.2 | No association between strength of faith and ED symptoms  Association between ED symptoms and:  -conformity  +value of achievement  +endorsement of self-enhancement/power | |
| Legenbauer et al. Germany | 2018 | Peer-reviewed journal article | Relationship between maladaptive core schemas, dysfunctional ED cognitions + binge-eating in EAs | | Weak | Quantitative | Cross-sectional | BN 100% female  BED 100% female  NC 100% female  Race/Ethnicity NR | 29  31  30 | | 28.1 ± 7.9 (TS) | Early maladaptive schemas:  BN + BED > NC  BN=BED  Mediated relationship: schemas relating to impaired autonomy / achievement -> cognitions about eating and loss of control -> craving intensity  schemas relating to impaired disconnection -> cognitions about eating and loss of control -> craving intensity  schemas relating to exaggerated vigilance -> cognitions about eating and loss of control -> craving intensity |  |
| Lucas USA | 2010 | Unpublished dissertation | Relationship between parental factors + ego development + disordered eating in EAs | | Strong | Quantitative | Cross-sectional | 100% female  NC  Uni students Race/Ethnicity NR | | 131 | NR | Approach to eating associated with mother’s approach to eating + mother’s parenting style (authoritarianism)  Mother’s authoritarian parenting style became a non-significant predictor of eating approach when personal well-being was taken into consideration  No association between approach to eating + ego development | |
| Lydecker et al. USA | 2014 | Peer-reviewed journal article | Relationship between white guilt + ED symptoms in EAs | | Moderate | Quantitative | Cross-sectional | 100% female  NC Uni students 53% White 21% Black 26% NR | | 374 | 19.1 ± 1.6 | TS:  Disordered eating associated with trait guilt  White group:  Bulimia associated with white guilt  Negative affect moderated relationship between white guilt + hunger, drive for thinness + bulimia  Distress tolerance moderated the association between white guilt + disinhibited eating, and drive for thinness | |
| Marta-Simoes & Ferreira Portugal | 2018 | Peer-reviewed journal article | Relationship between early memories of peer warmth / safeness, self-compassion and social safeness + ED symptoms in EAs | | Moderate | Quantitative | Cross-sectional | 100% female  NC  Occupation NR Race/Ethnicity NR | | 387 | 21.6 ± 1.7 | ED symptoms negatively associated with body appreciation + BMI  Effect of early memories of warmth + safeness on ED symptoms mediated by self-compassion + social safeness + body appreciation  Effect of self-compassion + social safeness on ED symptoms mediated by body appreciation | |
| Mugoya et al. USA | 2018 | Peer-reviewed journal article | Relationship between disordered eating, depressive symptoms, alcohol use + suicidality in EAs | | Weak | Quantitative | Cross-sectional | 81% female NC Uni students 79% White 11% Black 10% Other | 1598 | | 21.3 ± 5.5 | Disordered eating associated with suicidality + depressive symptoms  Interaction between disordered eating + alcohol use associated with depressive symptoms |  |
| Palladino et al. USA | 2013 | Peer-reviewed journal article | Impact of transition to EA on ED symptoms in EAs with + without T1D | | Strong | Quantitative | Longitudinal | T1D Uni students Male + female (% NR)  T1D  Non-uni Male + female (% NR)  NC  Uni students Male + female (% NR)  NC Non-uni Male + female (% NR)  Race/Ethnicity T1D sample: 96% Not Hispanic 2% Hispanic 2% NR  NC sample: 96% Not Hispanic 3% Hispanic 1% NR | | 88  29  91    31 | NR  NR  NR    NR | Uni students (T1D + NCs): no change in drive for thinness between T1 and T2  Drive for thinness decreased for non-uni students with T1D but increased for non-uni NC  No change in bulimia in any group | |
| Rawana et al. Canada | 2016 | Peer-reviewed journal article | Relationship between depressive + ED symptoms in male + female EAs | | Moderate | Quantitative | Cross-sectional | Females NC  Uni students  Males  NC Uni students  Ethnicity (TS): 37% White  35% Asian  28% Other | | 473  135 | 19.8 ± 2.3 (TS) | In female group, ED symptoms (restrained eating + emotional eating + external eating) associated with depressive symptoms  In male group, ED symptoms (restrained eating only) associated with depressive symptoms | |
| Shagar et al. Australia & Malaysia | 2019 | Peer-reviewed journal article | Compare the relationship between peer, family + media influence, thin-ideal internalization, body dissatisfaction + ED symptoms in Australian + Malaysian EAs | | Moderate | Quantitative | Cross-sectional | Australian 100% female  NC  88% uni students 81% White  9% Asian 10% Other  Malaysian 100% female  NC  86% uni students Race/Ethnicity NR | | 421    399 | 20.9 ± 3.4    20.6 ± 2.1 | In both Australian + Malaysian groups, ED symptoms associated with:  + Peer/ family / media influence  + Thin-ideal internalization  + Body dissatisfaction  Association between thin ideal + body dissatisfaction stronger in Australian group than Malaysian group  Association between family influence + internalization of thin ideal was significant for Malaysian but not Australian women | |
| Thurston et al. ^a^ USA | 2018 | Peer-reviewed journal article | Relationship between perceived stress, resilience + binge-eating in EAs | | Moderate | Quantitative | Cross-sectional | 100% female NC  Uni students 49% White  27% Black 23% Other | | 297 | 19.2 ± 1.5 | Binge-eating associated with:  +BMI  -Resilience  +Perceived stress  Significant interaction between perceived stress + resilience | |
| Waszczuk et al. ^a^ Norway | 2019 | Peer-reviewed journal article | Contribution of environmental + genetic factors to maintenance and co-occurrence of ED symptoms from adolescence to EA | | Moderate | Quantitative | Longitudinal | 57% female NC  Occupation NR Race/Ethnicity NR | | 1453 | 19.6 ± 2.0 | Maintenance of ED symptoms from adolescence to EA largely under genetic influence, with modest to moderate non-shared environmental influences.  Latent and time-specific genetic and environmental influences for drive for thinness and body dissatisfaction correlated more than with bulimic symptoms | |
| West et al. ^a^  USA | 2019 | Peer-reviewed journal article | Relationship between overweight / obesity, body dissatisfaction, dieting, weight-related teasing, food insecurity + binge-eating in high + low SES EAs | | Weak | Quantitative | Longitudinal | Low SES NC 55% female Occupation NR 50% White 27% Asian 23% Other  High SES  NC  50% female Occupation NR 81% White 10% Asian 9% Other | | 1187      992 | NR  NR | In both low + high SES groups, binge-eating during EA is predicted by adolescent:  +Overweight / obesity  +Dieting  Strength of relationships greater in high SES group than low SES group.  In high SES group only, binge-eating during EA is predicted by adolescent:  + Body dissatisfaction  + Family weight-related teasing  In low SES group binge-eating during EA is predicted by adolescent food insecurity  Friend weight-related teasing did not predict binge-eating in either group | |
| Treatment |  |  |  | |  |  |  |  | |  |  |  | |
| Brown et al. UK | 2016 | Peer-reviewed journal article | Compare feasibility + acceptability of FREED service model to TAU | | Moderate | Quantitative | Longitudinal | ED receiving FREED 96% female Occupation NR Race/Ethnicity NR  ED receiving TAU  98% female Occupation NR Race/Ethnicity NR | | 51  89 | 20.7 ± 2.5  20.5 ± 2.0 | Wait-times for assessment + treatment: FREED < TAU | |
| Dimitropoulous et al. Canada | 2013 | Peer-reviewed journal article | Clinicians’ perceptions of barriers / facilitators of effective transition from child to adult ED services | | Strong | Qualitative | Cross-sectional | % Female NR  ED HCPs Race/Ethnicity NR | | 23 | NR | Timing of transition from child to adult ED services should be determined by “readiness”, not age  Potential facilitators:  Educating parents about developmentally appropriate support  Supporting patient development of autonomy  Coordinated medical follow-up with primary physician prior to leaving paediatric services | |
| Javier & Belgrave USA | 2018 | Peer-reviewed journal article | Barriers + facilitators of ED treatment-seeking in Asian American EAs | | Moderate | Qualitative | Cross-sectional | Female  “At risk” for ED Occupation NR 100% Asian | | 26 | 19.3 ± 0.8 | Facilitators of treatment-seeking:  Available resources  Familial support  Barriers to treatment-seeking:  Lack of available resources  Stigma | |
| Koskina & Schmidt UK | 2019 | Peer-reviewed journal article | Single case-study of the treatment of an 18-year-old female with recent onset AN | | Strong | Quantitative | Longitudinal | Female  AN Occupation NR Race/Ethnicity NR | | 1 | 18 | ED symptoms reduced from 4.5 at assessment to 0.5 at 5-month FU.  Psychological distress reduced from 19 at assessment to 4 at 5-month FU  Participants outlined identity exploration work as the most helpful for recovery | |
| McClelland et al. UK | 2018 | Peer-reviewed journal article | Assess clinical outcomes in FREED patients + carers  Compare service utilization + BMI change in FREED patients + TAU patients | | Moderate | Quantitative | Longitudinal | ED receiving FREED  96% female  Occupation NR Race/Ethnicity NR  ED receiving TAU 98% female Occupation NR Race/Ethnicity NR  Carers Sex/Gender NR Occupation NR Race/Ethnicity NR | | 56  86  19 | 20.4 ± 2.4  20.4 ± 2.0  NR | Significant improvement in ED symptoms + other patient / carer outcomes between baseline, 3-months + 6-month assessments. Smaller improvement between 6- and 12-months.  Service utilization: FREED (100%) > TAU (74%)  BMI: FREED AN > TAU AN | |
| Weigel et al. Germany | 2014 | Peer-reviewed journal article | Compare duration of untreated ED in adolescents, EAs + adults | | Weak | Quantitative | Cross-sectional | EAs with EDs Female  Occupation NR  Adols. with EDs Female  Occupation NR  Adults with EDs Female  Occupation NR | | 25  19    14 | 21.3 ± 2.5  15.7 ± 2.9    33.7 ± 7.3 | DUI: EAs > adolescents (significance NR) | |

Abbreviations: Adols = adolescents; AN = anorexia nervosa; BED = binge eating disorder; BES = Binge Eating Scale; BMI = body mass index; DEPS-R = Diabetes Eating Problem Survey Revised; DUED = duration of untreated eating disorder; DUSC = duration of time until specialist contact; DUI = duration of illness; EAs = emerging adults; EAT-26 = Eating Attitudes Test; EDE-Q = Eating Disorder Examination Questionnaire; EDI-R = Eating Disorder Inventory Revised; EDs = eating disorders; HCPs = healthcare professionals; NC = non-clinical; NR = not reported; NES = night eating syndrome; RSA = respiratory sinus arrythmia; SES = socioeconomic status; TAU = treatment as usual; TS = total sample; T1D = type one diabetes.

^a^ Study is represented in two sections of the table (e.g. prevalence and aetiology)
